# Editorial: Identification, risk stratification, and optimized management for Lynch Syndrome

**DOI:** 10.3389/fonc.2023.1223568

**Published:** 2023-06-08

**Authors:** Inge Bernstein, Christina Therkildsen, Toni T. Seppälä

**Affiliations:** ^1^ Department of Surgical Gastroenterology, Aalborg University Hospital, Aalborg, Denmark; ^2^ Department of Clinical Medicine, Aalborg University, Aalborg, Denmark; ^3^ Gastro Unit, Copenhagen University Hospital, Hvidovre, Denmark; ^4^ Faculty of Medicine and Health Technology, Tampere University and TAYS Cancer Centre, Tampere, Finland

**Keywords:** Lynch Syndrome, mismatch repair genes, identification, surveillance, personalized medicine

## Introduction

Lynch Syndrome (LS) is caused by a pathogenic variant in one of the mismatch repair (MMR) genes, leading to cancers in various organs, including the colorectum (CRC), endometrium (EC), ovaries (OVC), stomach, small bowel, biliary tract, pancreas, and urinary tract (UTC). For decades, there has been a focus on defining, diagnosing, and treating LS, particularly since the discovery of the first MMR genes responsible for LS in the early 90s. Now, a new era is underway, focusing on personalized medicine.

This Research Topic aims to explore potential approaches to improve the identification of LS patients, technologies for individualized cancer risk estimation to guide more personalized cancer treatment and surveillance programs, and issues focusing on the quality improvement of established surveillance programs.

To achieve these goals, invitations to submit papers were sent through the Frontiers platform and via email to well-established colleagues in the LS research community. Thirteen articles — four reviews, seven original papers, and two case reports — were accepted for publication after review, all addressing various objectives within the scope.

## Identification and overall clinical aspects

Historically, research on LS has focused on CRC, followed later by EC. Well-established surveillance programs for these cancer types have been implemented, improving prognosis. Consequently, cancer-related deaths in LS patients are more frequently caused by other extracolonic LS-associated cancers (1).

In this Research Topic, Zalevskaja et al. demonstrated improved survival after pancreaticobiliary cancers in LS compared to sporadic cancer, although prognosis was still poor. Pancreatic and biliary tract cancers are rare tumour types among LS carriers, and Zalevskaja et al. call for more studies on molecular and immune profiles to learn about LS-associated pancreaticobiliary cancers’ suitability to possible immunotherapy.

Williams et al. present the landscape of current practice and future perspectives in the clinical management of LS patients in a minireview, touching on LS diagnostics, risk estimates, surveillance strategies around the world, and treatment options.

Strategies for identifying LS patients have evolved over time, from using clinical Amsterdam and Bethesda criteria towards guidelines recommending testing all newly identified CRCs for deficient MMR (dMMR) by MMR protein immunohistochemistry (IHC) or by microsatellite instability (MSI). Recently, recommendations have been extended to include all endometrial cancers and other extracolonic cancers known in LS. Cost-effectiveness analyses vary in different studies and are often country-specific. However, due to enhanced detection of LS and prognostic implications, universal testing of adenocarcinomas in several tumour types is recommended by numerous professional societies, including the National Comprehensive Cancer Network (NCCN), Society of Gynecologic Oncology (SGO), ACOG, and European Society for Medical Oncology (ESMO).

## Tumour analysis as an initial screening tool for LS identification

Concordance between such screening methods can vary for different tumour types. This variation is not necessarily important in universal testing of newly diagnosed cancers to identify LS, but caution should be taken when using abnormal IHC-guided single-gene genetic testing in CRC, EC, and OVC to identify LS patients, as this method may miss 8% of patients with LS according to Pan et al..

Moreover, a new potential screening tool is presented in this Research Topic, which may be relevant for screening of extracolonic cancers. Rasmussen et al. demonstrated that LS-associated UTC frequently exhibits loss of MMR protein expression, and they found no significant differences between IHC and a sequencing-based MSI approach using 54 markers.

## Risk stratification

Traditionally, carriers of any pathogenic variant in an MMR gene were thought to have a comparable risk of developing a range of different malignancies without distinction to the affected gene. Establishment of international LS databases, such as the Prospective Lynch Syndrome Database (PLSD) and the International Mismatch Repair Consortium (IMRC) database, and several other studies have improved the understanding that cumulative incidences of cancers and survival in LS-associated cancer patients are associated with the specific gene affected (1–3). The differences in risk may be explained by molecular variation in the driver mutations (4, 5). Even within each MMR gene or a specific variant, the cancer risk can still vary from <10 to >80% suggesting unknown genetic, epigenetic or environmental risk factors (3).

The review by Andini et al address the mild phenotype of *PMS2* carriers, specifically pointing out the low risk of CRCs that paradoxically may have more in common, biologically, with sporadic CRC as behaving more aggressive with a worse prognosis than LS-CRC induced by defects in *MLH1*, *MSH2* or *MSH6*.

LS is the most common cause of inherited CRC, but for women with LS, EC is most likely the sentinel cancer unless surgically prevented. Underkofler and Ring suggest, based on recently updated literature on identification and management of gynecologic cancers in LS, that gynecologists should be allowed to identify and prevent such cancers to improve prognosis in light of treatment changes towards more molecular classification and targeted therapy.

The use of molecular analysis to classify EC have also important clinical implication according to Riedinger et al (2023), who demonstrated that EC with epigenetic MMR defects have a poorer outcome that is independently associated with lymph node metastases. They therefore suggest implementation of MMR status and hypermethylation preoperatively for risk stratification of the EC patients.

## Colorectal cancer surveillance and outcomes

The adenoma-to-carcinoma sequence has traditionally been considered the pathway to CRC, leading to the recommendation of colonoscopy and polypectomy as optimal cancer prevention methods in international guidelines. However, the appropriate length of surveillance intervals and the degree of individualization in tailoring programs remain debated.

Approximately 6% of Lynch Syndrome (LS) patients undergoing colonoscopy surveillance exhibit multiple adenomas. Jain et al.show that the majority (87%) of these patients had advanced neoplasia or CRC, indicating that multiple adenomas represent a high-risk phenotype independent of genotype. This finding suggests that the presence of multiple adenomas should be considered when developing recommendations for individualized CRC surveillance.

Jamizadeh et al. also supports personalized surveillance intervals that take individual risk factors into account. Their findings showed that 35% of surveillance-detected CRC cases were found after 24 months, and thus outside their recommended biennial surveillance program. Additional factors, such as *MLH1* and *MSH2* pathogenic variants, male sex, current or previous smoking status, and high BMI were associated with an elevated risk of developing CRC and might be considered when setting the surveillance intervals.

Carmen et al. did, however, not find any statistically significant differences in the likelihood of developing adenomas, advanced adenomas, or CRC between carriers of variants in the four MMR genes. However, they observed a higher or earlier incidence of adenomas in carriers of *MSH2/EPCAM* variants and a non-significant tendency of increased risk among Hispanics. Consequently, they advocated for individualized screening programs and further research.

Many authors of this Research Topic emphasize the need for future prospective studies and, based on their findings, more individualized guidelines for LS management.

## New treatment approaches

Immune checkpoint-based therapy (ICT) has proven effective in managing solid microsatellite instability-high (MSI-H) tumours, regardless of organ site. LS patients may be optimal candidates for ICT, as most cancers in LS exhibit deficient mismatch repair (dMMR), MSI, and immune response activation. Though molecular differences have been observed between sporadic and LS MSI cancers, a systematic literature review found no difference in response rates between LS and sporadic MSI cancer patients (6).

Atiq et al. presented a case report of an LS patient with locally advanced prostate cancer who achieved significant tumour reduction after treatment with ICT in combination with androgen deprivation. Another case report by Liu et al. described an LS patient with adenocarcinoma in the rectum and prostate, followed by undifferentiated sarcoma in the neck, who achieved tumour regression and stable disease after ICT in combination with chemotherapy.

It is important to emphasize that data are still scarce, and the majority of studies presenting positive treatment response may indicate a selection bias towards publication, particularly in case reports. Therefore, more studies, preferentially from large clinical trials, are needed to evaluate the outcome of ICT in LS.

## Prevention by vaccination

The new era of precision oncology, based on tumour molecular profiling to tailor personalized treatment and immune system modulation, has transformed cancer prevention approaches for at-risk individuals. MMR deficiency in LS carriers leads to the accumulation of mutations in coding microsatellites, giving rise to frameshift peptides (FSP) recognized by the immune system as neoantigens. Cancer vaccines composed of commonly recurring FSP neoantigens have been evaluated in mouse models (7).

In a comprehensive review, Sei et al. (2023) discuss advances, technologies, and prevention strategies for the clinical translation of personalized risk-tailored vaccination strategies for LS carriers.

## Perspectives of the future

Despite the improved management of LS carriers in recent decades, there is still a need for research on studies addressing cancer development, prevention, and geographical differences to improve the prognosis for LS patients.

In summary, this Research Topic offers a comprehensive overview of the current state of LS research, highlighting recent advances and future directions in identification, surveillance, and treatment. The growing emphasis on personalized medicine holds promise for more effective management of LS patients, ultimately leading to improved outcomes and quality of life for those affected by this genetic syndrome.

## Author contributions

All authors listed have made a substantial, direct, and intellectual contribution to the work and approved it for publication.
